# The impact of graduated compression stockings on calf-vein deformation and blood velocity in patients awaiting total knee arthroplasty

**DOI:** 10.1186/s12891-021-04603-z

**Published:** 2021-08-23

**Authors:** Zaikai Zhuang, Yexian Wang, Yao Yao, Ying Shen, Dongyang Chen, Qing Jiang

**Affiliations:** 1 Nanjing Drum Tower Hospital Clinical College of Nanjing Medical University, Nanjing, 210008 People’s Republic of China; 2 State Key Laboratory of Pharmaceutical Biotechnology, Division of Sports Medicine and Adult Reconstructive Surgery, Department of Orthopedic Surgery, Nanjing Drum Tower Hospital, The Affiliated Hospital of Nanjing University Medical School, 321 Zhongshan Road, Nanjing, 210008 Jiangsu People’s Republic of China

**Keywords:** Graduated compression stockings, Vein diameter, Blood velocity, Venous thrombosis, Thromboprophylaxis

## Abstract

**Objectives:**

This study was designed to explore venous deformation of the lower extremities and the changes in venous hemodynamics in supine position before and after wearing graduated elastic stockings in patients awaiting total knee arthroplasty (TKA).

**Method:**

The leg veins of 21 elderly patients awaiting TKA were imaged in the supine position with and without knee-length graduated compression stockings (GCS) according to a fixed protocol. Measured parameters including the lateromedial (LM) diameter, anteroposterior (AP) diameter, and cross-sectional area (CSA) of the great saphenous vein (GSV), gastrocnemius vein (GV), soleus vein (SV), posterior tibial vein (PTV), fibular vein (FV), and anterior tibial vein (ATV). In addition, the mean and maximum velocities of the popliteal vein (PV) and superficial femoral vein (FSV) were measured.

**Results:**

GCS-related compression was observed for all the measured veins. Maximal reduction was observed for the GV and SV, whereas the GSV exhibited the lowest degree of GCS-related compression. The mean cross-sectional area reduction values associated with GCS were 33.1 ± 41.2 % for the GSV, 94.8 ± 11.1 % for the GV, and 85.6 ± 20.3 % for the SV, while the mean reduction of anteroposterior diameter was 18.1 ± 34.5 % for the GSV, 89.0 ± 22.5 % for the GV, and 72.9 ± 35.1 % for the SV, and the mean reduction of the lateromedial diameter was 25.9 ± 36.4 % for the GSV, 89.6 ± 19.6 % for the GV, 78.2 ± 28.3 % for the SV. No significant GCS-related changes in blood velocity in the superficial femoral veins or popliteal veins were detected.

**Conclusions:**

For elderly patients awaiting TKA, knee-length GCS can significantly reduce calf vein dilation while at rest in the supine position, with the greatest reductions being observed for the soleus and gastrocnemius veins. These data might help provide a theoretical basis for the GCS in reducing incidence of deep vein thrombosis in patients undergoing TKA.

## Introduction

Deep vein thrombosis (DVT) is defined as the occurrence of a blood clot within a deep vein, most often in the legs. Thrombus formation primarily occurs in the context of immobilization, the rate of DVT following knee arthroplasty ranges from 41 to 85 % without thromboprophylaxis [1, 2]. In-hospital mortality rates for patients suffering from venous thrombosis are roughly 1.1 %, and these patients experience mortality rates of 9.4 % over a 6-month follow-up period [3]. Some prior reports have suggested that up to 50 % of patients may develop post-thrombotic syndrome (PTS) following DVT, potentially reducing their disease-specific quality of life [4, 5]. Preventing DVT following knee arthroplasty is thus essential.

The intermuscular vein thrombosis is the most common distal lower limb thrombus [6]. And we and others have previously reported that soleus vein (SV) dilatation is an independent predictor of DVT risk following major orthopedic surgery [7, 8].

Mechanical thromboprophylaxis is most commonly achieved by using graduated compression stockings (GCS), as wearing GSC has been shown to significantly reduce the risk of DVT in orthopedic surgery patients [9]. Some reviews have found that GCS may decrease DVT incidence by reducing venous diameters and increasing rates of venous blood flow [10, 11]. So we would like to observe whether GCS can eliminate the risk factor for intermuscular vein dilation in patients undergoing TKA.

This study was designed to analyze lower extremity venous deformation before and after wearing graduated elastic stockings in patients awaiting TKA, with a focus on the soleus and gastrocnemius veins. The overall goal of this study was to help provide a theoretical basis for the GCS in reducing incidence of deep vein thrombosis in patients undergoing TKA.

## Materials and methods

All methods were carried out in accordance with the Declaration of Helsinki.

The hospital Ethics Committee approved the present study, and all patients provided informed consent. In total, 21 patients awaiting TKA were analyzed. Patients were excluded from this study if they presented with comorbidities including severe peripheral neuropathy, severe lower extremity arterial disease, severe lower limb deformities, thrombosis, sensory impairment, and visible varicose veins. Patients were additionally excluded if they were excessively obese (BMI ≥ 40), excessively underweight (BMI < 18.5), or had undergone venous surgical procedures.

### Experimental protocols

All measurements were made in a room with a comfortable environment. Patients were instructed to lay in the supine position with both of their legs being exposed. A pillow (8 cm in height) was placed ~ 5 cm above the popliteal fossa to allow for ease of measurement. To ensure that the same vein segment in the same position was measured, three marking lines were made on the skin with a non-water-soluble black ink pen. Lines were located at the midpoint of the thigh, the knee joint space, and the midpoint of the calf.

Patients were allowed to rest for 15 min until their blood pressure and heart rate stabilized, after which baseline measurements of the lower limb were made. For these measurements, the great saphenous vein, anterior tibial vein, posterior tibial vein, and fibular vein were assessed at the midpoint of the calf, the popliteal vein was assessed at the knee joint space, and the superficial femoral vein was assessed at the midpoint of the thigh. The gastrocnemius vein and soleus vein were measured at the largest diameter of the vein via ultrasound, with the distance between the vein and the probe being recorded and the skin being marked with a pen to ensure that subsequent scans were made at the same location. Prior studies have utilized vein diameter as a metric for characterizing decreases in venous cross-sectional area, which would suggest that the vessel cross-section remained roughly circular. However, we found that vein cross-sectional geometry was roughly elliptical before and after compression. As such, we measured the lateromedial diameter, anteroposterior diameter, and cross-sectional area of each vein to more accurately reflect venous deformation. The mean and maximum blood velocity values for the superficial femoral vein and the popliteal vein were additionally recorded.

After patients put on the compressive stockings, they were allowed to rest for an additional 15 min prior to analysis. To ensure appropriate visibility of the veins across the stockings, a sufficient amount of gel was applied such that the underlying tissue was wet. A new set of duplex measurements was then made for each vein through the compressive stockings. Superficial femoral vein and popliteal vein scans were taken above the proximal edge of the stocking. Care was taken to avoid applying additional pressure to the limb with the transducer, as this had the potential to alter the venous diameter.

### Compression stockings

Knee-length gradient elastic compression stocking size was selected using the measuring chart recommended by the manufacturer (Medical Supplies Pty Ltd, Haoshide, China). The stockings used for this study were of an elastic knit with 16–22 mmHg of compression pressure (compression class I). Studies have reported that with respect to postoperative thromboprophylaxis, there were no differences between the knee-length, low-pressure GCS and GCS of other sizes or pressures, whereas patients report feeling more comfortable when wearing knee-length, low-pressure GCS [12].

### Doppler ultrasound measurements

**Fig. 1:**
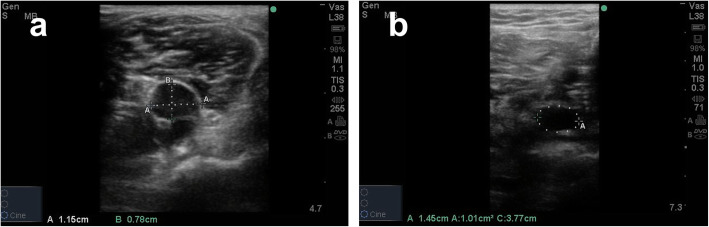
**a** Line A represents the lateromedial diameter of the vein and line B represents the anteroposterior diameter of the vein. **b** The cross-sectional area of the vein

**Fig. 2:**
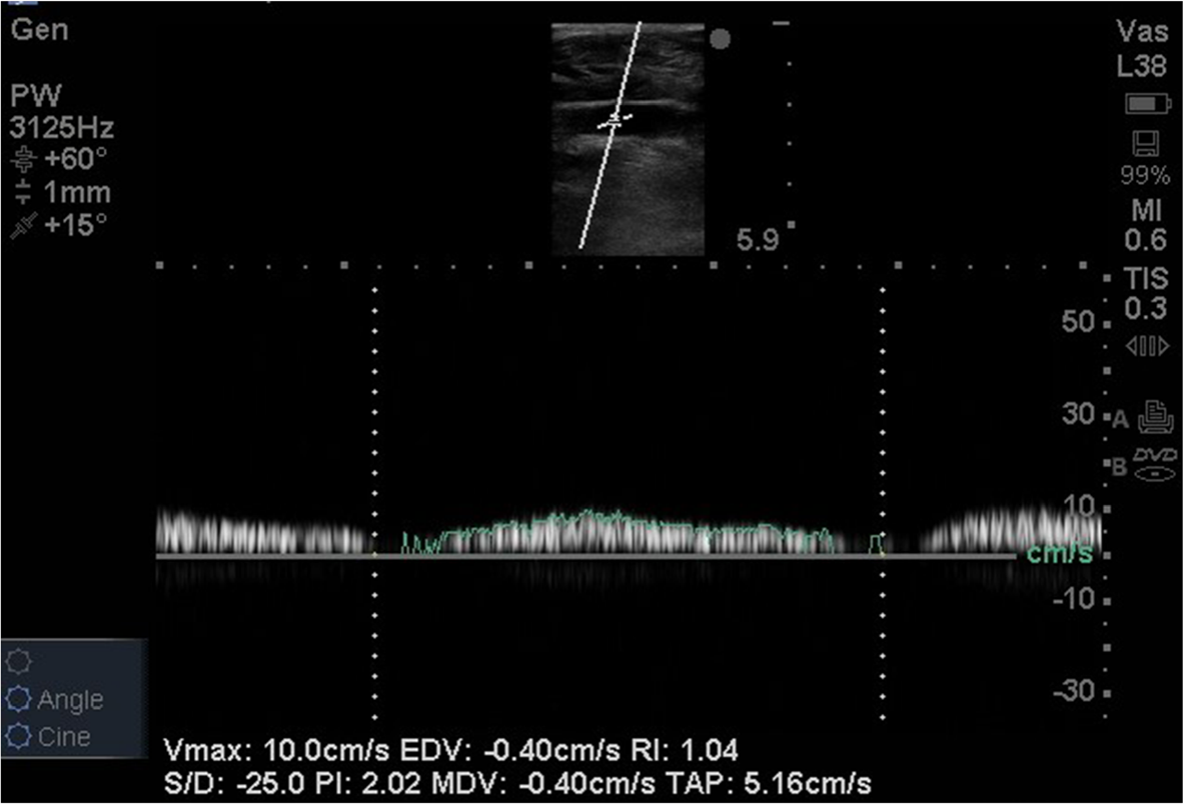
The Doppler sample volume cursor was placed in the center of the vein, and mean blood velocity and maximum blood velocity were calculated

A Sonosite M-Turbo ultrasound system was used for all 2D transverse scans of the veins. After appropriate scans were complete, images were frozen and the anteroposterior diameter, lateromedial diameter, and cross-sectional area of each vein were measured (Fig. 1). Longitudinal scans were obtained for the superficial femoral vein and the popliteal vein. The Doppler sample volume cursor was placed in the center of this vein, and the mean blood velocity and maximum blood velocity were calculated (Fig. 2). Mean values for these three measurements were made.

### Statistical analysis

SPSS v. 26 (IBM Corp., Armonk, NY, USA) was used for all statistical testing. Data are given as means with standard deviations (SD), and were analyzed via Wilcoxon rank-sum test. *P* < 0.05 was the significance threshold for this study.

## Results

In total, 21 patients were included in the present study with a mean age of 64 ± 7 years (range: 54–76 years). The mean body mass index (BMI) of these patients was 26.6 ± 3.4 kg/m^2^ (range: 21.3–35.1 kg/m^2^), and 17/21 patients were female.

**Fig. 3:**
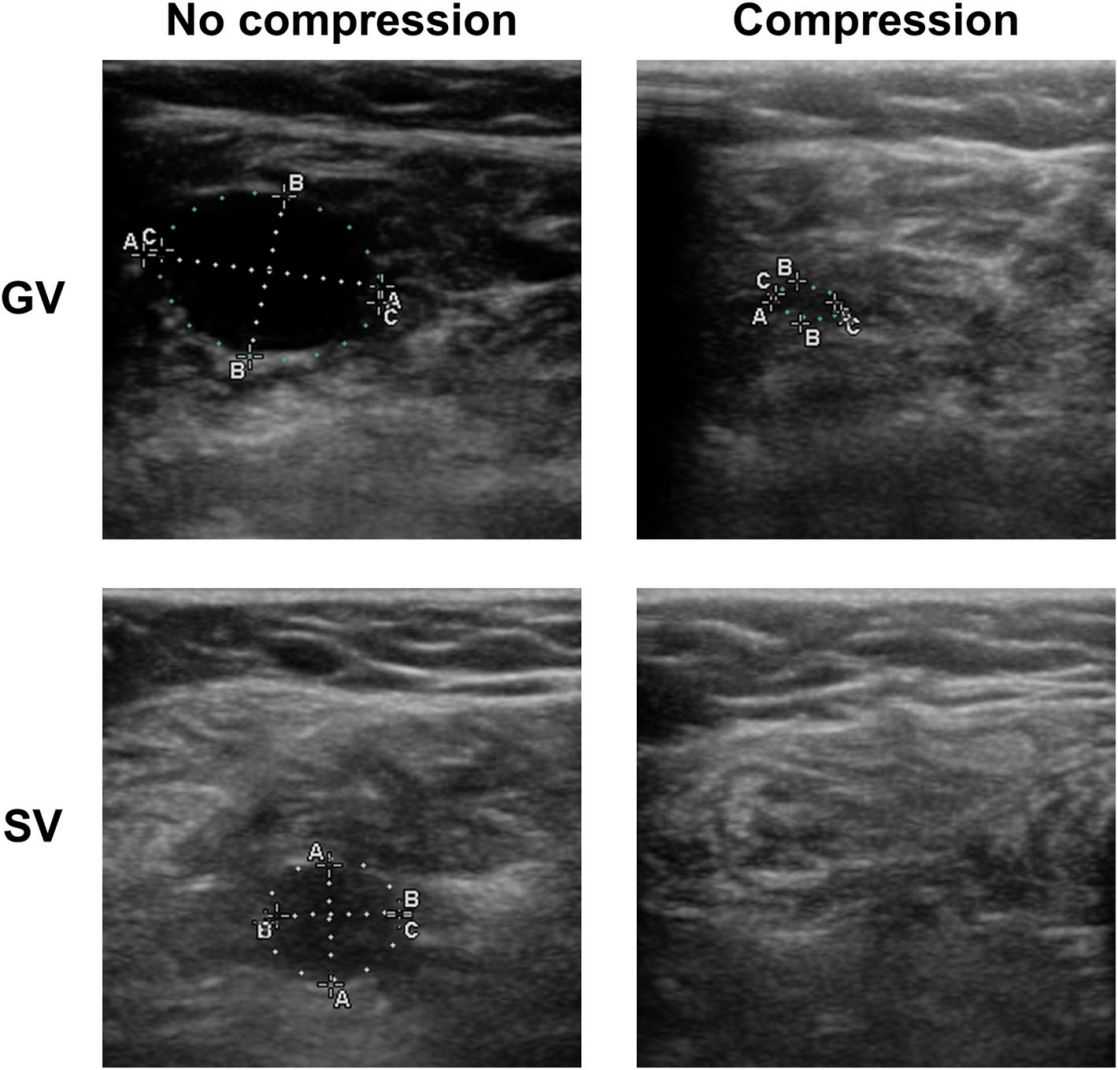
Duplex sonography imaging of gastrocnemius veins and soleus veins, with and without compression. **A** = lateromedial diameter, **B** = anteroposterior diameter

Wearing GCS was associated with the compression of most analyzed veins including the GSV, GV, SV, PTV, FV, and ATV. Maximal reduction was observed for the GV and SV, whereas the GSV exhibited the lowest degree of reduction. Gastrocnemius veins were closed in 16 of these 21 patients, while soleus veins were closed in 12 of these 21 patients in the context of GCS-mediated compression. Representative images of GV and SV deformation are shown in Fig. 3.

Cross-sectional areas and diameters of the calf muscle veins in these patients are shown in Table 1. The GV exhibited mean uncompressed and compressed cross-sectional area values of 21.5 ± 25.1 mm^2^ and 2.4 ± 6.2 mm^2^, respectively (*P* < 0.001). Similarly, GCS were associated with a significant reduction in GV diameter from 5.1 ± 3.1 mm to 0.9 ± 2.1 mm (AP; *P* < 0.001) and 4.4 ± 2.3 mm to 0.7 ± 1.6 (LM; *P* < 0.001). The SV also exhibited significant GCS-related reductions in cross-sectional area in these patients (40.3 ± 25.0 mm^2^ vs. 5.0 ± 7.3 mm^2^; *P* < 0.001). GCS additionally decreased the diameter of the SV from 8.1 ± 2.5 mm to 2.1 ± 2.7 mm (AP; *P* < 0.001) and 5.8 ± 2.0 mm to 1.3 ± 1.7 (LM; *P* < 0.001). The mean cross-sectional area reductions for the GV and SV were 94.8 ± 11.1 % and 85.6 ± 20.3 %, respectively.

**Table 1:** The deformation of the calf muscle veins in compressed states

	Uncompressed	Compressed	*P*-value	% Reduction
GV CSA (mm^2^)	21.5 ± 25.1	2.4 ± 6.2	< 0.001	94.8 ± 11.1
GV diameter AP (mm)	5.1 ± 3.1	0.9 ± 2.1	< 0.001	89.0 ± 22.5
GV diameter LM (mm)	4.4 ± 2.3	0.7 ± 1.6	< 0.001	89.6 ± 19.6
SV CSA (mm^2^)	40.3 ± 25.0	5.0 ± 7.3	< 0.001	85.6 ± 20.3
SV diameter AP (mm)	8.1 ± 2.5	2.1 ± 2.7	< 0.001	72.9 ± 35.1
SV diameter LM (mm)	5.8 ± 2.0	1.3 ± 1.7	< 0.001	78.2 ± 28.3

Data are means ± SD

*GV *gastrocnemius vein, *SV *soleus vein, *CSA *cross sectional area, *AP *anteroposterior, *LM *lateromedial

Cross-sectional area values and diameters for all deep veins measured in the present study are shown in Table 2. A significant GCS-related reduction in the cross-sectional area of the ATV was observed in these patients (4.2 ± 2.4 mm^2^ to 1.1 ± 1.0 mm^2^; *P* <0.001), and this coincided with a compression-mediated decrease in ATV diameter from 1.9 ± 0.6 mm to 0.8 ± 0.7 mm (AP; *P* < 0.001) and 3.0 ± 0.9 mm to 1.0 ± 0.9 (LM; *P* < 0.001). The cross-sectional area of the PTV similarly differed in a GCS-related manner (15.4 ± 12.3 mm^2^ vs. 4.8 ± 3.7 mm^2^; *P* < 0.001), and the PTV diameter declined from 4.3 ± 2.0 mm to 2.5 ± 0.8 mm (AP; *P* < 0.001) and 3.9 ± 1.6 mm to 2.3 ± 1.3 (LM; *P* < 0.001) when patients wore GCS. GCS application reduced the cross-sectional area of the FV in these patients from 44.0 ± 19.2 mm^2^ to 14.2 ± 11.4 mm^2^ (*P* < 0.001) and reduced the diameter of the FV from 8.6 ± 2.3 mm to 3.9 ± 2.3 mm (AP; *P* < 0.001) and 6.2 ± 1.7 mm to 3.4 ± 2.6 (LM; *P* = 0.001). The mean CSA reductions for the ATV, PTV, and FV were 69.5 ± 29.8 %, 59.0 ± 28.2 %, and 65.2 ± 30.5 %, respectively.

**Table 2:** The deformation of the deep veins in compressed states

	Uncompressed	Compressed	*P*-value	% Reduction
ATV CSA (mm^2^)	4.2 ± 2.4	1.1 ± 1.0	< 0.001	69.5 ± 29.8
ATV diameter AP (mm)	1.9 ± 0.6	0.8 ± 0.7	< 0.001	57.6 ± 39.2
ATV diameter LM (mm)	3.0 ± 0.9	1.0 ± 0.9	< 0.001	62.7 ± 33.3
PTV CSA (mm^2^)	15.4 ± 12.3	4.8 ± 3.7	< 0.001	59.0 ± 28.2
PTV diameter AP (mm)	4.3 ± 2.0	2.5 ± 0.8	< 0.001	35.1 ± 31.0
PTV diameter LM (mm)	3.9 ± 1.6	2.3 ± 1.3	< 0.001	37.5 ± 31.9
FV CSA (mm^2^)	44.0 ± 19.2	14.2 ± 11.4	< 0.001	65.2 ± 30.5
FV diameter AP (mm)	8.6 ± 2.3	3.9 ± 2.3	< 0.001	50.0 ± 35.3
FV diameter LM (mm)	6.2 ± 1.7	3.4 ± 2.6	0.001	48.4 ± 38.7

Data are means ± SD

*ATV *anterior tibial vein, *PTV *posterior tibial vein, *FV * fibular vein, *CSA *cross sectional area, *AP *anteroposterior, *LM*  lateromedial

Cross-sectional area and diameter values for superficial veins analyzed in the present study are shown in Table 3. GCS application was associated with a significant reduction in the GSV cross-sectional area from 5.1 ± 3.4 mm^2^ to 3.2 ± 2.6 mm^2^, (*P* = 0.002) and in GSV diameter from 2.8 ± 1.0 mm to 2.3 ± 1.2 mm (AP; *P* < 0.001) and 2.1 ± 0.8 mm to 1.4 ± 0.6 (LM; *P* = 0.003). The mean GCS-related cross-sectional area reduction for the GSV was 33.1 ± 41.2 % in these patients.

**Table 3:** The deformation of the great saphenous vein in compressed states

	Uncompressed	Compressed	*P*-value	% Reduction
GSV CSA (mm^2^)	5.1 ± 3.4	3.2 ± 2.6	0.002	33.1 ± 41.2
GSV diameter AP (mm)	2.8 ± 1.0	2.3 ± 1.2	< 0.001	18.1 ± 34.5
GSV diameter LM (mm)	2.1 ± 0.8	1.4 ± 0.6	0.003	25.9 ± 36.4

Data are means ± SD

*GSV *great saphenous vein, *CSA *cross sectional area, *AP *anteroposterior, *LM *lateromedial

The peak blood velocity in the femoral vein of all 21 patients without GCS was 16.1 ± 3.9 cm/s, and it did not significantly increase in these patients with GCS (16.6 ± 4.3 cm/s; *P* = 0.339). Similarly, no GCS-related increases in mean blood velocity values in the femoral vein were observed (9.9 ± 3.7 cm/s vs. 10.7 ± 4.2 cm/s; *P* = 0.068) (Table 4).

**Table 4:** Blood velocity in the femoral and popliteal vein with and without knee-length GCS

	Uncompressed	Compressed	*P*-value
PV peak flow velocity (cm/sec)	9.5 ± 3.0	10.1 ± 3.5	0.227
PV mean flow velocity (cm/sec)	5.1 ± 2.0	5.3 ± 2.5	0.414
FSV peak flow velocity (cm/sec)	16.1 ± 3.9	16.6 ± 4.3	0.339
FSV mean flow velocity (cm/sec)	9.9 ± 3.7	10.7 ± 4.2	0.068

Data are means ± SD

*PV *popliteal vein, *FSV *superficial femoral vein

The peak blood velocity in the popliteal vein of all 21 patients without GCS was 9.5 ± 3.0 cm/s, and it did not significantly increase in these patients with GCS (10.1 ± 3.5 cm/s; *P* = 0.227). Similarly, no GCS-related increases in mean blood velocity values in the popliteal vein were observed (5.1 ± 2.0 cm/s vs. 5.3 ± 2.5 cm/s; *P* = 0.414) (Table 4).

## Discussion

The present study was designed to explore the GCS-related deformation of lower extremity veins and associated hemodynamic changes in patients awaiting TKA in the supine position. Through these analyses, we found that GCS were able to significantly compress most measured veins including the GSV, GV, SV, PTV, FV, and ATV, with the effect being most pronounced for the GV and SV. No significant changes in blood velocity in the superficial femoral or popliteal veins were observed.

Our results are consistent with those of multiple prior studies, which have demonstrated that GCS can significantly compress calf muscle veins. However, the percentage of mean vein reduction in this study was higher than in previous reports. Lord et al. [13] observe effective compression of both superficial and deep veins by GCS when patients were in the supine position, with this reduction being superior for the calf muscle veins. The mean internal diameter of the uncompressed and compressed SV in their study was 2.20 mm and 0.86 mm, respectively, with a 60.9 % mean reduction in the context of GCS-mediated compression. Arcelus et al. [14] found that the use of compressive stockings was associated with a mean reduction in the medial GV cross-sectional area of 54.5 % with a corresponding 30 % reduction in the lateral gastrocnemius vein cross-sectional area. Smith et al. [15] analyzed patients undergoing abdominal or neck surgery and found that the median GV diameter fell significantly from 2.6 mm to 1.6 mm following GCS application during surgery. Furthermore, Jeanneret et al. [16] determined that GCS were sufficient to significantly compress the GV as measured through compression stockings in the prone position. In the present report, we found that some calf muscle veins were not closed in analyzed patients. This may be attributable to the fact that we utilized class 1 elastic compression GCS with a compression pressure of 16–22 mmHg, or it may be due to differences in the locations at which these calf muscle veins were measured.

GCS have also been reported to effectively compress deep veins. For example, in their study of patients in the supine position, Lord et al. found that the PTV internal diameter fell from 2.83 mm without GSC to 1.69 mm with GCS, with the FV internal diameter similarly being reduced significantly from 3.51 mm to 1.92 mm [13]. Downie et al. [17] further found that the cross-sectional area of the PTV and FV were significantly reduced by elastic compression stockings for individuals in the prone position.

GCS can similarly suppress superficial veins, although we detected less pronounced compression of these superficial veins relative to that of the calf muscle veins and deep veins in the present study. This may be because interface pressure can drive a rise in intramuscular pressure, whereas the subcutaneous pressure is solely driven by the GCS [18]. However, Partsch et al. [19] observed nearly equivalent compression of superficial and deep veins by GCS, with 3 of 9 analyzed patients exhibiting more pronounced superficial vein compression as compared to that observed for deep veins.

Unexpectedly, no significant changes in femoral or popliteal vein blood velocity were detected as a function of GCS-mediated compression. However, this is consistent with some prior reports. For example, Giron et al. [20] and Kaori et al. [21] similarly determined that knee-length GCS had no significant impact on peak flow velocity values in the femoral or popliteal veins. Paul et al. [22, 23] and Keith et al. [24] further determined that thigh-length GCS had no significant impact on popliteal or femoral vein blood velocity for patients in the supine position. Such findings are not, however, universal. Jamieson et al. [25], for example, determined that GCS were able to significantly increase the mean blood flow velocity of the common femoral vein in pregnant women in the immediate postnatal period. Another study conducted by Espeit et al. [26] further determined that GCS were sufficient to increase the popliteal venous blood velocity of patients in the prone position. These inconsistencies may be attributable to differences in subject posture, GCS class, GCS size, the duration of GCS use, and/or the duration of immobility among studies.

We acknowledge that there are a number of limitations to the present study. First, the sample size was relatively small. However, this factor does not affect the conclusion of the paper due to the results of 21 patients revealed high consistency and the power values we calculated have exceeded a minimum threshold of 0.8 [27]. So, we considered that our data are convincing. Second, ultrasound-based analyses can be operator-dependent, although the same trained vascular sonographer conducted all measurements in this study to minimize this form of bias. Third, we only focused on knee-length GCS and did not explore the possible efficacy of compression stockings that extend above the knee. Lastly, measurements in the present study focused solely on the supine position, and additional analyses of other body positions (including standing and sitting) would better reflect the effects of GCS-mediated compression in real-world situations.

## Conclusions

In summary, the results of this study indicated that knee-length GCS can significantly reduce calf vein dilation in elderly patients awaiting TKA while in the supine position at rest, with the greatest reductions being observed for the soleus and gastrocnemius veins. These results have the potential to help provide a theoretical basis for the GCS in reducing incidence of deep vein thrombosis in patients undergoing TKA.

## Data Availability

The datasets used and analyzed during the current study are available from the corresponding author on reasonable request.

## Abbreviations

TKA: Total knee arthroplasty
GCS: Graduated compression stockings
LM: Lateromedial
AP: Anteroposterior
CSA: Cross-sectional area
CSA: Great saphenous vein
GV: Gastrocnemius vein
SV: Soleus vein
PTV: Posterior tibial vein
FV: Fibular vein
ATV: Anterior tibial vein
PV: Popliteal vein
FSV: Superficial femoral vein
DVT: Deep vein thrombosis
PTS: Post-thrombotic syndrome
BMI: Body mass index
